# Revisiting out-of-pocket requirements: trends in spending, financial access barriers, and policy in ten high-income countries

**DOI:** 10.1186/s12913-018-3185-8

**Published:** 2018-05-18

**Authors:** Thomas Rice, Wilm Quentin, Anders Anell, Andrew J. Barnes, Pauline Rosenau, Lynn Y. Unruh, Ewout van Ginneken

**Affiliations:** 1 University of California, Los Angeles, USA; 2 Berlin University of Technology, Berlin, Germany; 3 Lund University, Lund, Sweden; 4 Virginia Commonwealth University, Richmond, USA; 5 University of Texas Health Science Center, Houston, USA; 6 University of Central Florida, Orlando, USA; 7 European Observatory on Health Systems and Policies, Berlin University of Technology, Straße des 17. Juni 135, 10623 Berlin, Germany

**Keywords:** Access, Coinsurance, Copayments, Deductibles, Cost-sharing, Comparative health systems, Out-of-pocket costs

## Abstract

**Background:**

Countries rely on out-of-pocket (OOP) spending to different degrees and employ varying techniques. The article examines trends in OOP spending in ten high-income countries since 2000, and analyzes their relationship to self-assessed barriers to accessing health care services. The countries are Australia, Canada, France, Germany, the Netherlands, New Zealand, Norway, Sweden, Switzerland, the United Kingdom, and the United States.

**Methods:**

Data from three sources are employed: OECD statistics, the Commonwealth Fund survey of individuals in each of ten countries, and country-specific documents on health care policies. Based on trends in OOP spending, we divide the ten countries into three groups and analyze both trends and access barriers accordingly. As part of this effort, we propose a conceptual model for understanding the key components of OOP spending.

**Results:**

There is a great deal of variation in aggregate OOP spending per capita spending but there has been convergence over time, with the lowest-spending countries continuing to show growth and the highest spending countries showing stability. Both the level of aggregate OOP spending and changes in spending affect perceived access barriers, although there is not a perfect correspondence between the two.

**Conclusions:**

There is a need for better understanding the root causes of OOP spending. This will require data collection that is broken down into OOP resulting from cost sharing and OOP resulting from direct payments (due to underinsurance and lacking benefits). Moreover, data should be disaggregated by consumer groups (e.g. income-level or health status). Only then can we better link the data to specific policies and suggest effective solutions to policy makers.

## Background

All countries rely on out-of-pocket (OOP) spending to help fund their health care systems. OOP spending includes both direct payments made for uncovered services (due to lack of insurance or lacking benefits) and cost sharing requirements such as coinsurance and deductibles. It serves two main purposes: as a source of revenue, and to help reduce demand for services. However, controversy surrounds OOP payments and opponents typically voice two concerns. Charging people for their medical care means that those with the greatest need, and those with the lowest income, will feel the brunt. Moreover, patients may forgo necessary care - in contrast to other forms of financing such as taxes and premiums, which cannot be avoided by forgoing health care.

In recent years there is a perception that in many countries costs are being shifted to patients. Policy interest, therefore, is high, but there has been little cross-country research on actual trends in OOP spending or on consumer perceptions of reductions in access that result from higher OOPs. The purpose of this article is to: (a) systematically assess trends in OOP spending in ten prominent high-income countries as well as trends in people’s perception of any resulting impediments regarding accessing needed medical care; and (b) discuss some of the policies responsible for these trends. As part of this effort, we propose a conceptual model for understanding the key components of OOP spending.

The four most relevant previous studies published since 2010 are presented in Table 1 [1–4]. The one most similar to the current study [4], by Zare and Anderson, examines four of the ten countries in the current study, although in the U.S. it examines only the Medicare program and has a somewhat shorter time period (2000–2010). Our study differs from previous ones in several important ways that extends our understanding of OOP payments trends across countries. Most previous research considers OOP spending only in terms of cost sharing policies. This overlooks the important role of direct payments for uncovered services, which in some countries may play a larger role in OOP spending, as does the complicated interplay with Voluntary Health Insurance. Such insurance strongly affects levels of OOP spending. In addition, this study examines recent changes in OOP policy in each country. Moreover, unlike previous research, we examine the financial barriers of OOP spending with regard to reducing access to care. Overall, the study attempts to link three things: aggregate trends in spending, perceived barriers to access, and governmental health policies.

**Table 1:** Recent Previous Research on Out-of-Pocket Spending

Study	Countries	Data/Variable	Key Findings	Relevance
Baird, 2016 [1]	Australia, France, Israel, Japan, Poland, Russia, Slovenia, Switzerland, United States	Individual survey on OOP spending compared to income from Luxembourg Income Study (2010 for most countries)	• In median country, 13% of people spend more than 10% of income in OOP. • Varies from 3% (France) to 17% (Switzerland). • Poor and elderly at greatest risk of cata-strophic spending.	• Focuses on percentage of population with high OOP spending during a single year. • Emphasizes groups that are most financially vulnerable. • Does not examine countries’ health policies. • Does not examine perceived barriers on access to care.
Palladino et al., 2016 [2]	Austria, Belgium, Czech Republic, Denmark, France, Germany, Netherlands, Spain, Sweden, Switzerland	Survey of people age 50 and older from Health, Ageing and Retirement in Europe, with data on changes in OOP spending and experiencing catastrophic OOP spending (30% or more of income), from 2006/7 to 2013 (Great Recession)	• Very large range in changes in OOP spending (− 11% in Netherlands to + 101% in Austria). • Increase in catastrophic spending: from 2.3 to 3.9% over study period. • People age 50 and older spent more in 8 of11 countries. • Countries do provide financial protection for poor.	• Focuses on changes in OOP spending during limited time period. • Does not examine countries’ health policies. • Does not examine perceived barriers on access to care.
Tambor et al., 2011 [3]	27 countries in the European Union	Review of international data bases, laws and regulations, and reports on changes in patient cost sharing requirements since 1990	• Cost-sharing requirements vary a great deal between countries, and have increased significantly in many. • Tax-based systems more likely to use co-payments, insurance-based systems more likely to use deductibles and coinsurance. • Almost all countries have policies to protect the poorest and/or sickest.	• Focuses on health policies in countries, but little detail provided. • Includes extremely diverse set of countries. • Does not examine perceived barriers on access to care.
Zare & Anderson, 2013 [4]	France, Germany, Japan, United Kingdom, United States (Medicare only)	Various data sets from OECD, WHO, European Observatory, and country-specific reports, time period 2000–2010; separately examine cost sharing for pharmaceuticals, inpatient, and ambulatory care	• Inflation-adjusted OOP spending, and spending divided by income, increased in all countries. • Percent of total national health care paid OOP declined in most countries due to protection mechanisms for poor and/or sick.	• Focuses on health policies in 5 countries. • Does not examine perceived barriers on access to care.

## Methods

### Choice of countries and time period

Our choice of countries was based on the data available for self-reported access barriers due to OOP spending. To assess these barriers, we use a long time-series of household survey data collected by the Commonwealth Fund. This survey now includes data from 11 countries: Australia, Canada, France, Germany, the Netherlands, New Zealand, Norway, Sweden, Switzerland, the U.K., and the U.S. For the quantitative analysis of trends in spending and perceived access barriers, we include all 11 countries. When describing policy changes since 2000, however, we exclude New Zealand because of the difficulty of obtaining accurate historical policy data. The European Observatory for Health Systems and Policies regularly publishes updated books on developments in health policy in its Health Systems in Transition series. While the other countries’ books have all been updated since 2011, New Zealand’s book dates back to 2001 [5].

The study time frame varies for different parts of our study. When examining aggregate data from the OECD and Commonwealth Fund, we consider the date range from 2000 to the most recent time period. When examining details about how OOP costs have changed in the individual countries, data back to 2000 was not always available. Therefore, we focused on an approximately 10-year period, beginning in 2005 for most countries.

### Conceptualizing out-of-pocket costs

#### Definition of terms

This study conceptualizes OOP spending as the sum of cost sharing requirements for covered or insured services, direct payments for uncovered/uninsured services, and informal payments. It does not include premium payments. Because informal payments are negligible in our set of countries, we do not consider them further. There are three issues, however, that complicate implementing this definition.

**Fig. 1:**
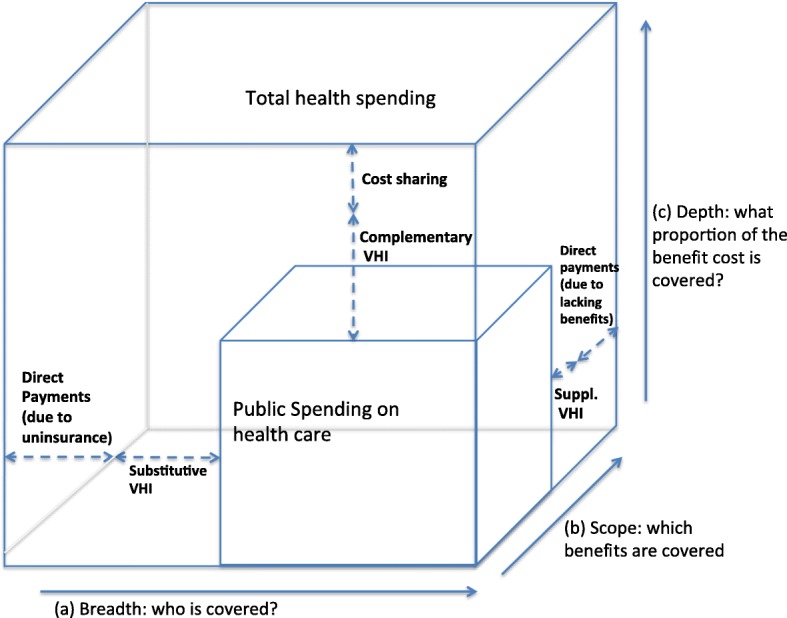
Three dimensions determine the level of out-of-pocket spending. Adapted from Busse and Schlette, 2007 [7]

The first concerns the use of aggregate annual OOP spending vs. spending for an individual service. Later in the article, we show that in many of the ten countries examined here, inflation-adjusted, aggregate OOP *spending* has not risen very much over the past decade. This is sometimes assumed to mean that OOP expenses are not a growing problem. This is not correct, however. If cost sharing requirements increase, people may respond by using fewer services, which obscures the true impact of growing cost sharing requirements because lower usage will result in lower aggregate OOP payments. The more appropriate way to examine whether people are facing an increase in the financial barriers that result from OOP payments is to examine how coverage and benefit policies as well as cost sharing requirements are changing over time, not how much aggregate OOP payments are changing. This is discussed in more detail below and presented graphically in Fig. 1.

A second issue relates to disentangling the role of voluntary health insurance (VHI). These products serve a variety of purposes, depending upon country. The most basic and common forms include supplementary VHI, which pays for services that are not covered by the national health plan (e.g., dental care), and complementary VHI, which pays for cost-sharing requirements for services covered in the national plan (if allowed under national legislation, as it is often seen as undermining the utilization-reduction effect). In some countries, VHI may also serve as a primary (“substitutive”) insurance coverage scheme. Finally, VHI sometimes can help patients get access to care without waiting as long as others who are obtaining public care but who lack VHI. If services are paid for by VHI, they do not constitute OOP spending. But lower OOP spending does not necessarily mean lower financial barriers, as it may be more costly in premiums to purchase VHI than to simply pay the OOP.

Finally, we do not examine OOP spending for long-term care (LTC), for two reasons. First, the countries examined have very different LTC schemes with widely diverging rules regarding patient responsibility for LTC costs, such that it was not possible to summarize these requirements without a large number of caveats. Second, LTC costs, to a large extent, fall only on one segment of the population – the elderly.

#### Analytic framework

With these issues in mind, we propose an analytical framework that is based on the three-dimensional cube that was first proposed by Busse et al. 2007 [6, 7] and which was subsequently adopted by the WHO in their World Health Report 2010 [8]. The 2007 framework captures the three dimensions of population coverage (breadth), scope of services (depth) and level of coverage (cost-sharing). Our revision has added the level of OOP and VHI to these dimensions (see Fig. 1). This allows us to systematically analyze the specific sources and components of OOP payments as well as the role of VHI.

The three dimensions, along with related policies, together determine the level of OOP. The first dimension is about who is covered (*breadth*). OOP payments may arise as a result of direct payments due to lack of insurance. The second dimension concerns which services are covered (*scope*). In this case OOP payments occur as direct payments resulting from lacking benefits/underinsurance. The third dimension relates to what proportion of benefit cost is covered (*depth*). Here, OOP payments may occur if cost-sharing schemes apply that require insured/covered individuals to pay a share of the benefit cost. As an important subcategory of OOP payments, cost sharing may take several forms. The most well-known are direct methods (copayment, coinsurance, and deductibles) and indirect methods (e.g. extra billing, reference pricing).

In the presentation of the study results regarding “Country-Specific OOP Policies,” below, we employ these concepts of breadth, scope, and depth in presenting current policy and trends. Ideally, to fully understand these policies and trends, one would conduct separate analysis of how three determinants of OOP spending have changed over time in each of the ten countries, but that is beyond the scope of this article and likely would fill a lengthy monograph.

#### Objective trends and perceived access barriers

One of the purposes of this study is to determine if there is a relationship between objective, aggregate OOP spending and perceptions of barriers to access. While there is certainly face validity to the hypothesis - if people have to spend more per service, economic theory would tell us they will use less - there are reasons for suspecting that this sort of causal relationship may be mitigated.

In the typical international definition, which we employ here, premiums are not counted towards OOP spending. [9] But premiums, of course, can reduce the use of needed services. In countries without universal coverage, like the U.S., premiums form a major barrier towards obtaining coverage [10]. Moreover, through an income effect, high premiums reduce disposable income, making less income available for purchasing health care services. A similar scenario relates to taxes. Payroll deductions for health care coverage, which are common in many social insurance countries, also reduce disposable income. Finally, these same issues apply to VHI: depending on how visible VHI premium payments are to the individual, they may also be viewed as a barrier to accessing care.

Moving away from definitional concerns, perceived access burden is likely to be affected by distributional issues that are difficult to observe in aggregate data. Countries that have a relatively low maximum on individual OOP expenditures are likely to see these costs spread out more equally among the population, meaning that fewer people will perceive cost-related access problems. In contrast, if there are high or even non-existent maximum spending thresholds, then some (sicker) people will be subject to higher spending and are more likely to experience access burdens. More broadly, a country’s social safety net is an overarching determinant of perceived access burdens.

### Data sources and analysis

OECD data on OOP spending, from the years 2000 to the most recent available, are used to show aggregate trends in such spending by country. In addition, longitudinal, country-specific consumer survey data from the Commonwealth Fund are used as the source of perceived financial access problems. Sample sizes ranged from 1000 to over 7000, depending on country.

However, much of the study data come from individual country sources (e.g. legislation, national reports). Specifically, we sought information on current levels and changes in both cost sharing requirements and direct payments for uncovered services. One primary source were the book series, *Health Systems in Transition,* published by the European Observatory on Health Systems and Policies. [11] Country-specific sources are also used when discussing the policy modifications that were responsible for changes in OOP spending.

Linear regressions were used to test whether trends in OOP spending differed across countries categorized to have historically low OOP costs with higher recent growth, historically moderate OOP costs with lower recent growth, or historically high OOP costs with low to no growth in recent years. In the analyses, the probability of making a type I error was set to 0.05.

## Results

### Aggregate trends in OOP spending by country

**Fig. 2:**
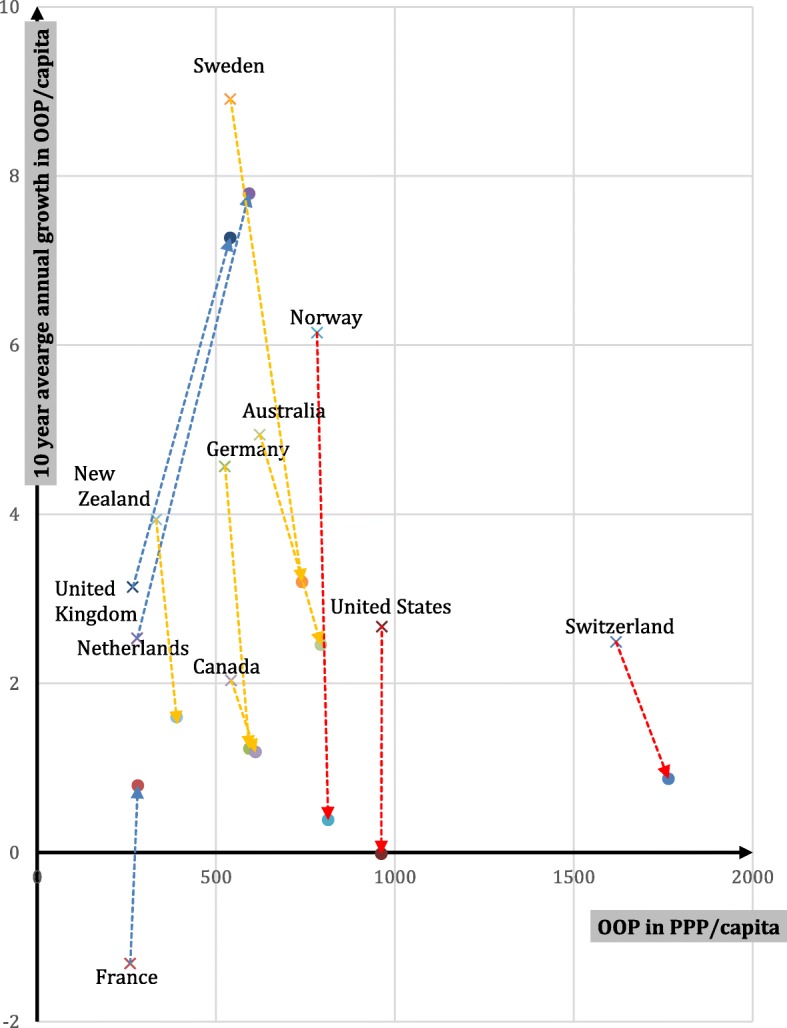
OOP spending per capita (in PPP and constant prices) vs growth in OOP per capita (in PPP and constant prices), from 1994-2004 vs. 2004-2014. Note: The base of each line, represented by “x,” shows the average annual rate of increase in PPP-adjusted per capita in OOP spending during the 1994-2004 period. The end of each line, represented by the arrowhead, provides the same figure for the 2004-2014 period. Countries are combined into three groups. The blues lines represent countries with historically low OOP spending; the red, countries with historically high OOP spending; and the yellow, countries with mid-level historical OOP spending. Source: adapted from OECD Health Statistics, 2017 [12]

Comparing OOP spending data over time and between countries is very difficult due to frequent breaks in the data, as well as differing definitions and variations in reporting between countries. We aim to compensate in part for these limitations by focusing on longer, successive, ten-year intervals rather than year-on-year increases, the latter often showing skewed results due to changes in data collection methodology. Figure 2 plots OOP spending per capita in 2004 and 2014 (in PPP and constant prices) versus growth rate averages in two previous 10 year intervals (i.e. time periods 1994–2004 and 2004–2014). The beginning of each line, represented by “x,” shows the average annual rate of increase in PPP-adjusted per capita in OOP spending during the 1994–2004 period. The end of each line, represented by the arrowhead, provides the same rate for the 2004–2014 period.

The figure suggests that there are three logical groupings of countries. First, countries where historically OOP spending has been lower compared to the others (UK, Netherlands, France) and which have experienced higher recent growth rates (2004–2014 compared to 1994–2004). The second group consists of countries in which OOP spending is in the middle range (Sweden, Australia, Germany, Canada); they have experienced a decreasing rate of growth when comparing the period 1994–2004 with 2004–2014, but OOP payments are still increasing. Finally, the third group consists of countries that have consistently experienced the highest OOP spending (Norway, United States, Switzerland), with some growth in 1994–2004, but little change in spending between 2004 and 2014. Thus, the more countries spent on OOP payments, the lower the growth rate. It is important to be aware that the reported figures are in constant US$ purchasing power parities (PPP). This is useful if one wants to compare OOP spending patterns over time and across countries. However, the fact that in some countries there was little to no increase in constant US$PPP does not mean that there was no increase in spending when expressed in the local currency or that no extra cost-sharing requirements were implemented. For example, although Norwegian OOP spending growth in the period 2004–2014 seems minimal in Fig. 2, OOPs per capita increased from approximately 5740 to about 8215 when expressed in Norwegian Krone (NOK) [12].

To confirm that the three groups did indeed experience divergent trends depicted in Fig. 2, a linear regression model was used. The results of this test, shown in Table 2, indicate that the three groups of countries indeed differed in their growth rate in OOP costs per capita in the decade prior to 2004 compared to the decade that followed. Specifically, those with historically low OOP costs but higher recent growth experienced an average *increase* of 3.63% (group 1), while those with historically moderate OOP costs with lower recent growth *declined* (group 2, − 3.00%, *p* < 0.001), as did those with historically high OOP costs with low to no recent growth (group 3, − 3.56%, *p* < 0.001). There were no significant differences in growth rates between these latter two country groups.

**Table 2:** Differences in Growth in Out of Pocket Costs Between Country Groupings

	Predicted Mean Percent Change in OOP/Capita 1994–2004 vs. 2004–2014	*p*-value
Group 1 - Historically low OOP costs with higher recent growth	3.63	reference group
Group 2 - Historically moderate OOP costs with lower recent growth	−3.00	< 0.001
Group 3 - Historically high OOP costs with low to no recent growth	−3.56	< 0.001

These trends illustrate our first key finding: a gradual convergence in OOP spending between countries. The convergence, of course, is slow in coming, since from a low base even a large increase in the percentage growth constitutes a relatively small increase in dollar or euro spending, when compared to low growth from a high base.

### Country-specific OOP policies

In this section, we analyze changes in the barriers imposed by OOP payments by examining how policy modifications affect the breadth, scope, and depth of coverage over the last decade. For an overview of current policies in each country except New Zealand, refer to the Table 1 in Appendix, and for changes in such policies over the past decade, Table 2 in Appendix. Here we will focus on the changes between 2004 and latest available year. We discuss the countries according to the three groups as identified in Fig. 2.

### Group 1: Historically low OOP costs, higher recent growth (France, Netherlands, UK)

OOP spending in France is the lowest among the ten countries and has remained fairly stable in spite of some recent increases. For decades, France has had broad universal coverage of the population through statutory health insurance and near universal complementary coverage. This means that OOP does not occur as a result of lacking breadth or scope, but rather from cost-sharing requirements (depth). Nominal co-payments are charged for almost all physician services, hospital services, auxiliary services, prescription drugs, vision care, and dental care. Each type of health service has an annual cap of around €54, but these are additive. Generous exemptions from cost sharing payments as well as free complementary VHI (also through vouchers) exist for the poor, those with chronic illness, and women more than five months pregnant. Starting in 2010 all those with an annual income below €11,611 (reduced to €9825 in 2016) received vouchers to purchase complementary VHI. There is some extra billing for full-time public hospital doctors and a few other professional medical groups. In 2016 a major change in OOP charges was implemented: a daily hospital co-payment of about €18 for medical or surgical procedures that cost in excess €120. Many patients, however, are exempt from these out-of-pocket charges. Overall, growth in OOP seems to relate to small increases in cost sharing requirements in the form of co-payments.

Like France, the Netherlands historically has had very low per capita OOP spending. OOP spending in the Netherlands mostly is the result of direct payments due to changes in the benefit basket and various cost sharing requirements. Unfortunately, OOP spending data cannot be separated into cost sharing vs. direct payments due to the health care accounting practices in the Netherlands. Although there have been yearly changes in the benefit basket by adding or removing services and pharmaceuticals, it is nevertheless likely that increases in cost sharing requirements are responsible for changes in OOP spending, mostly as a result of rising deductibles. Starting in 2008 a €150 deductible was established (exempting GP care, maternity care, and care for children under 18), and it has increased more than 2.5 fold, to €385, in 2016. Moreover, the delisting of certain services (e.g. dental care for adults) may have had some effect on increasing direct payments, although the impact was mitigated by the high rates of participation in voluntary health insurance. Direct payments are required for some drugs, adult dental care, and some medical equipment, among other things. Indeed, in 2015, 84.1% of all insured purchased VHI, which typically covers dental care (73% of Dutch people) and physiotherapy [13].

Historically, the U.K. has not systematically excluded benefits (it rather provides services “to such extent as [considered] necessary to meet all reasonable requirements” [14]. This means that the relatively high growth in OOP (2nd after the Netherlands) in 2004–2014 must be mostly ascribed to increased cost sharing requirements. Still, per capita OOP spending has been very low by international standards, with only those in France spending less. Inpatient and outpatient services are received free at point of service in most cases, but there are co-payments for prescription drugs that have been growing and amounted to £8.40 per prescription in 2016. These co-payments were capped at £104 annually in 2009 and remain the same today. Furthermore, although the co-payment maximum was lowered in 2006 (from £384 to £189), there have been regular increases since then. Similarly, there are co-payments for dental services (the amount of which varies by service). Both drug and dental co-payments have risen by 2% per year since 2005. Children and students, those age 60 and above, people with specific medical conditions and those on low-income schemes are exempted from co-payments for drugs and dental services, and are covered for vision services (which is normally not covered). Unsurprisingly, the market for VHI is rather small. Individuals buy VHI to avoid waiting lists, have some choice over the physician they visit, and for more comfortable rooms [15].

### Group 2: Historically moderate OOP costs, lower recent growth (Australia, Canada, Germany, Sweden)

The Australian Medicare system provides universal population coverage. OOP occurs mostly due to a lack of scope of benefits, particularly dental benefits, and various cost sharing requirements. Indeed, although the system fully covers care in public hospitals, it requires co-payments for most other services as well as care in private hospitals. Per-service co-payments for GPs have grown considerably in recent years, rising from $AUD 11.8 (€8.02) in 2005 [16] to $AUD 29.56 (€20.10) in 2014 [17], a 2.5-fold increase, with most of the increase occurring since 2009. Other services that have experienced significant aggregate increases in OOP expenditures over recent years are prescription drugs, specialist care, and dental care. The timing of these changes appears to relate to a change in national government dating from 2013. The new government has been attempting to reduce national health outlays. Australia is unusual in that the government provides strong incentives for people to purchase private supplementary VHI, and close to half of the population does so. VHI typically covers the higher user charges of private hospitals, and helps reduce co-payments for other services. Significantly, however, it does not cover co-payments for GP services. Interestingly, a recent survey shows an emerging disillusionment with private insurers, as membership is declining and 75% of young adults considered downgrading or dropping their policy in the past 12 months [18].

The Canadian health system, governed by the Canada Health Act of 1984, provides universal population coverage. The benefit package includes inpatient and most outpatient care but excludes some important categories including outpatient prescription drugs, dental, or vision care. This results in substantial OOP due to direct payments, mostly for pharmaceuticals. It is difficult to generalize much beyond this because each of the 13 provinces and territories chooses the extent to which it covers such services. For example, each has its own pharmacy benefits program and formulary and the prevalence of complementary VHI coverage also varies. Complementary insurance covers many of the potential OOP costs and some provinces require employers to provide it for employees. Furthermore, beginning in 2010 most provinces adopted catastrophic income-based pharmacy insurance that protects individuals for catastrophic costs in purchasing medications. In almost all cases the OOP costs for the very poor are covered through a variety of federal and provincial programs. Since the number of changes has been limited, OOP has grown moderately in the period 2004–2014. Some provincial innovations stand out. Ontario is implementing publicly funded universal comprehensive drug coverage, called “pharmacare,” for children and youth.

The German system provides universal population coverage either through social health insurance (SHI) (almost 90% of the population) or substitutive VHI. As OOP policies of substitutive VHI vary widely (and data is largely unavailable), we focus on SHI. OOP spending in Germany is related to both direct payments for services not covered by SHI (e.g., over-the-counter drugs) and cost-sharing for SHI covered services, but the relative importance of cost-sharing versus direct payments is not known because of the health system’s accounting methods. OOP spending above reference prices for dental care and medical aids can be substantial; for example, reference prices for dental care cover only about 50% of the costs of standard care. Cost-sharing requirements increased in 2004 for such things as prescription drugs, inpatient care, and physician and dental care, while at the same time adult eyeglasses were excluded from coverage. Since these increased requirements became effective in 2004 they do not affect the average growth rate in the 2004–2014 period and therefore do not contradict the observed drop in average growth in 2004–2014 compared to the 1994–2004 period. The most plausible explanation for the low growth in this period is the fact that in 2013, co-payments for physician visits were discontinued. The most important protection mechanism against OOPs is the exemption of children under 18 years of age and a maximum cost-sharing limit of 2% of annual income (or 1% for patients with severe chronic conditions). This has remained largely unchanged over the study period.

Sweden’s health system provides universal population coverage for a broad basket of services. The majority of OOP is thus attributable to cost sharing requirements. These include co-payments for most health services adults use in Sweden, including hospitalization. Co-payments for health care visits and hospitalization vary across the 21 counties although policies have converged over time. Co-payments are differentiated to steer patients towards use of primary care during office hours. Co-payments for visits to specialist doctors without a referral are about twice as much as for a regular visit to a primary care doctor. In most counties children below age 20 are exempt from co-payments. There is a combined cap for each 12 month period determined at the national level, maximizing total co-payments for outpatient care. Deductibles, co-payments and caps for prescription drugs and dental care are determined fully at the national level. Dental care for individuals under age 22 is free. For adults, there is a deductible of SEK 1000 (€103) for prescription drugs followed by a stepwise increase in subsidies ending in a 12 months cap of SEK 2200 (€226). For dental care to adults, the deductible is SEK 3000 (€308), followed by increase in co-insurance to 85% above SEK 15,000 but without an overall cap. Additional minor changes in terms of additional subsidies for dental care to elderly were introduced in 2013. In 2009, prescription cost-sharing policy was changed such that patients now usually pay the full price for generic alternatives that are not the lowest cost generic. In 2012 cost-sharing limits on prescriptions as well as for outpatient services were increased (from SEK 1800 to SEK 2200 (€ 185–226) and SEK 900 to SEK 1100 (€ 92–113) respectively). These increased cost sharing requirements seem not to have had great impact on the average growth rate in 2004–2014, which was lower than in the previous period. This can perhaps be explained by the fact that the requirements were relatively minor and partially offset by better dental benefits.

### Group 3: Historically high OOP cost, low to no growth in recent years (Norway, Switzerland, United States)

Norwegians enjoy universal population coverage for a broad basket of benefits. OOP payments relate to cost sharing for most health care services with the exception of inpatient care. The majority of acute-care OOP payments are associated with outpatient care, dental care, and prescription drug use. Cost sharing requirements have not changed much in the past decade and OOP spending has remained at a stable yet high level. Requirements are set nationally for designated services and populations. For example, general practitioner and outpatient specialist co-payments were NOK 152/201 (€16/21) (depending on level of education of GP) and NOK 345 (€37) in 2017. Prescription drugs deemed essential and on an approved list called the “blue list” can cost as much as NOK 520 (€55) per prescription. Two different cost-sharing ceilings are set by Parliament annually. The first ceiling was NOK 2205 (€234) in 2017 and is a limit on cost-sharing amounts paid to physicians, psychologists, hospital care, radiology and blue-list drugs. The second ceiling was NOK 1990 (€211) in 2017 and limits OOP spending on physiotherapists, eligible dental care (identified conditions), some rehabilitation services, and treatment abroad (if referred by Oslo University hospital). Certain diseases (e.g. many communicable disease) and patients (e.g. children, the disabled, pregnant women) are subject to reduced or no cost-sharing while services like adult dental care face the highest cost-sharing.

The Swiss system provides universal population coverage for a broad basket of services. There have been some changes to cost-sharing requirements in Switzerland since the early 2000’s. In 2004, the minimum deductible increased from CHF230 (€210) to CHF300 (€275) and the maximum deductible increased in 2005 from CHF1500 (€1373) to CHF2500 (€2288). In 2006, coinsurance increased to 20% for brand drugs if a cheaper generic is available. Direct payments were affected by the exclusion of eye glasses from coverage in 2011, and the inclusion of complementary and alternative medicine into the benefits package in 2012. In fact, direct payments account for about 80% of all OOPs. Given the diversity of insurance plans in Switzerland the reliance on OOPs is also affected by changes in choices made by those purchasing coverage. The share of the population opting for insurance plans with higher deductibles (in exchange for lower premiums) has increased considerably over time. For example, the proportion with a deductible of more than CHF2000 (€1830), increased from less than 15% in 2009 to almost 23% in 2014. Over the same period of time, the proportion of the population opting for insurance plans with limited choice of providers increased from about 35% to about 62% as a result of similar attempts to exert downward pressures on premiums through benefit design.

The United States is the only country of the ten that has substantial OOP costs due to direct payments resulting from uninsurance. However, these costs have almost certainly gone down during the Obama administration because many people obtained coverage under the Affordable Care Act (ACA), which expanded the scope of the Medicaid program for the poor in many states and mandated people not covered elsewhere to insure themselves through insurance exchanges. As a result uninsurance among the adult population age 18 and older dropped from 17.1% in 2013 to 11.0% in 2016 [19]. (The rate is closer to 9% when the entire population is included since children’s uninsurance rates are lower.) Still, many Americans lack insurance coverage and pay for all costs out of pocket.

Unlike other high-income countries, Americans receive insurance benefits through a number of sources: employers, Medicare (seniors and the disabled), Medicaid (low-income persons and some disabled), and through individual coverage, some of which is purchased from private insurers in the ACA’s insurance exchanges. OOP payments are best considered within each of the main coverage types.

- Medicaid covers a very wide scope of services and this coverage is deep, with minimal cost sharing requirements. As noted, the breadth of program coverage has risen dramatically in recent years, particularly due to expansion under the ACA, doubling from 35 million people in 2000 to 70 million in 2016. One of the main problems is that physician payment rates are so low in some states that it is difficult for program enrolees to find primary care and specialist physicians to treat them.
- The breadth of Medicare coverage is nearly universal in the age 65+ population; the program covers many disabled people as well. Since 2006, with the implementation of prescription drug benefits, nearly all types of services are covered. Depth of coverage is relatively low with coinsurance rates as high as 20% for physician services and no out-of-pocket ceiling. As a result, 86% of Medicare beneficiaries have supplemental coverage [20] to pay for many coverage gaps. Sources include subsidized coverage from former employers, unsubsidized “Medigap” private insurance coverage (which is mainly complementary), and Medicaid for those with low incomes. Cost sharing requirements change only modestly year to year.
- Just over half of insured Americans receive coverage through employers. The scope of services covered tends to be broad. However, cost sharing requirements in such plans have risen steeply in recent years. The most dramatic changes have been for deductibles. On average, annual deductibles for employees who are only covering themselves have gone up almost 2.5-fold, from $602 in 2005 to $1478 in 2016. There have also been substantial increases in the maximum OOP costs beneficiaries can incur each year. In 2005, 33% of employees had a maximum of $3000 or more. But in 2016, this had risen to 66%. Depending on the insurer and type of service, employees are also subject to coinsurance or co-payments, but these requirements have been relatively stable over time except for brand-name drugs that are not on an insurer’s formulary. Most employees do not have coverage for dental care and vision services; for those that do, cost sharing requirements changed little over the past 10 years. There is no major market for complementary coverage. The main supplementary coverage is for dental care.
- Beginning in 2014, individuals have also obtained coverage on the ACA’s insurance exchanges. Deductibles are quite high in the most commonly purchased “Silver” plans, averaging almost $3600 in 2017 [21].

U.S. cost-sharing has been rising mostly in the employment-based markets. The reasons for rising deductibles are straightforward: they provide a way of moderating premium increases – which have risen much more slowly than deductibles. In view of these deductible increases, the most likely explanation for relatively stable aggregate OOPs shown in Fig. 2 is that OOP growth resulting mainly from deductible increases has been compensated (in the aggregate) by lower utilization. A second reason is that the expansion of Medicaid under the ACA has offset some the aggregate effects of the employer and individual market deductible increases since Medicaid has few cost-sharing requirements. Finally, most people who originally obtained coverage on the ACA exchanges were previously uninsured [22], so the ACA resulted in a net reduction in these people’s OOP spending.

### Perceived access problems and the role of OOP spending

Many factors determine whether people will report that they experienced a cost-related access problem. We focus on its relationship to levels and changes in OOP spending but other factors - not examined in this study - can be important as well. Earlier we mentioned expenses that are not included in the definition of OOP spending, such as insurance premiums and taxes, may affect responses to questions about perceived reduction in access to care. But beyond the definitional, individual characteristics such as current socioeconomic status, changes in that status, income distribution, employment, and country-specific differences in attitudes about access to care may also affect responses.

**Fig. 3:**
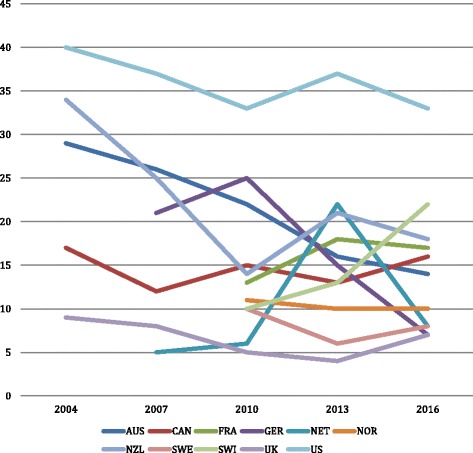
Percent adults reporting any of three access problems due to costs. Source: Commonwealth Fund, 2016 [29]

The Commonwealth Fund survey asks respondents about three aspects of perceived access, and compiles country-specific statistics on the percentage of adults who: (1) did not see a doctor when sick, (2) skipped a medical test or treatment recommended by a doctor, or (3) did not fill a prescription or skipped a dose because of cost in the past year. The data in Fig. 3 show the percentage of adult respondents who experienced any of these three things. Countries are grouped as they were in Fig. 2 (solid lines are the highest spending countries), dotted lines the lowest spending, and dashed lines the middle spending countries. In 2016, the U.S. had by far the highest rates of reported access problems, and the U.K. the lowest, with Germany, Netherlands and Sweden also low. We are particularly interested in country-specific trends over the study period to see if they correlate with changes in a country’s policies regarding what services are covered and, for covered services, cost sharing requirements. Thus, perceived OOP barriers within countries in each of the historical spending categories are described below to contextualize self-reported access problems in terms of national OOP spending trends.

### Group 1: Historically low OOP costs, higher recent growth (France, Netherlands, UK)

The most noteworthy pattern in Fig. 3 is the dramatic fluctuation in perceived barriers to access in the Netherlands. Only 6% of Dutch reported one of the access problems in 2010, but this almost quadrupled to 22% just three years later. Interesting, the burden fell back to historical levels (8%) by 2016. The surge between 2010 and 2013 is likely the result of the institution of deductibles and then their fast increase in deductibles. The highest year-to-year increase in deductibles took place between 2011 and 2013, when the deductible increased from €170 to €220 (2012) and to €350 (2013). When deductible growth had levelled off by 2016, the reported access barriers also reduced.

Perceived barriers were very low and reasonably steady in the U.K., consistent with residents’ low OOP spending responsibilities and lack of major changes in policy. It rose somewhat in France, but what is more surprising is that a country with such low OOP spending shows the third highest rate of perceived barriers in 2016. While the reasons for this are not clear, one possibility is that most OOP spending in France is for primary care services, which most people use. As noted, there are small co-payments required for physician, hospital, and auxiliary services, prescription drugs, and vision and dental care. The small increases in reported access problems in France between 2010 and 2016 likely relate to higher co-payments (i.e., the co-payment for hospital care in 2013). These trends may have led to reforms adopted in 2016 that will help poorer persons obtain complementary insurance policies. Starting in 2016 health insurance including complementary coverage was guaranteed to the poor in France free of charge. Those with low income will no longer have to prove employment to qualify for complementary VHI – three months of stable and regular professional activity or mere residence is sufficient [23].

### Group 2: Historically moderate OOP costs, lower recent growth (Australia, Canada, Germany, Sweden)

In line with country-level trends in OOP costs growth slowing, two countries in Group 2 had substantial decreases in self-reported access problems: Germany (between 2010 and 2016) and Australia (over the entire time period). The former may be the result of the discontinuation of co-payments for physician services in 2013. It is difficult, however, to explain the decline in perceived access problems in Australia. Since 2004 the reporting of access problems has fallen in spite of substantial increases in co-payments for GP, specialist, and dental services and pharmaceuticals. Perhaps the existence of VHI plays a role in protecting individuals from high OOP. However, enrolment rates have been relatively steady, at just under half of the population, since 2000.

Canada and Sweden have reported relatively few - and stable - cost-related access problems. This is in line with our observation that benefit baskets and cost sharing requirements in these countries have been fairly stable.

### Group 3: Historically high OOP cost, low to no growth in recent years (Norway, Switzerland, United States)

The country with the highest reported access problem is the U.S. This is not surprising given both high uninsurance rates and high levels of cost-sharing, particularly in employer-sponsored coverage. Nevertheless, reported access barriers declined considerably between 2004 and 2016. In the earlier period, a contributing factor for the decline was almost certainly the 20% rise in Medicaid enrolment between 2004 and 2016. More recently, it was a result of three major provisions of the ACA: the Medicaid expansion, premiums subsidies for those purchasing coverage on the exchanges, and requiring employers to allow children under the age of 26 to remain on parents’ family coverage plans.

Perceived access problems have continued to rise in Switzerland and reached the second highest value (22%) after the U.S. in 2016. Although Switzerland is the highest spender on OOP of all ten countries, *growth* in spending has been very low (see Fig. 2). The increased barriers therefore may have been the result of shifting OOP spending patterns. In fact, people in Switzerland are increasingly opting for high-deductible plans in exchange for lower premiums, which may create financial access problems for people with these plans.

The more striking observation is that Norway shows low access barriers in spite of OOP spending is the third highest of the ten countries. The Norwegians seem to achieve this by having relatively high co-payments coupled with relatively low ceilings. Low reported access barriers likely are the result of an equitable spread of OOP payments among the population. Moreover, the country enjoys a low unemployment rate and strong social protection mechanisms.

## Discussion

### Limitations

Any discussion of OOP spending across countries needs to be tentative for a variety of reasons, including data availability, quality and comparability, different roles of VHI across countries, and the fact that OOP requirements and utilization are intertwined such that – and as described earlier – aggregate trends mask what is occurring at the individual service level. Nevertheless, cross-country comparability can be enhanced by considering all relevant components of OOP spending along the three coverage dimensions (see Fig. 1), including cost sharing requirements, voluntary deductibles, direct payments for uncovered services, the population covered, and the interaction with VHI policy. That is the approach that we have taken here.

The data and analyses presented here are subject to several limitations. One concerns acquiring and summarizing data from the ten countries. Aggregate spending data suffer from frequent breaks in data collection methodology, which we have tried to control for by taking 10-year intervals. Frequent changes in cost-sharing requirements and in the design of benefit baskets complicate analyses and comparison of information across countries. Furthermore, many people and illnesses are exempt from cost sharing requirements, so changes reported usually apply only to a subset of the population.

The data on reported access problems from the Commonwealth Fund are also subject to various limitations discussed earlier. One of the most important is that the definition of OOP spending may not coincide with the things a person considers when reporting on cost-related access problems. For example, both premiums and taxes pose financial burdens but neither is defined as an OOP cost. Heightening this problem is the fact that different countries use these mechanisms to much different degrees. Second, the data are not broken down by survey respondents’ demographic characteristics. Third, response rates vary by country, and it is possible that they may be lower among vulnerable subgroups that may be more likely to experience access problems. A recent cross-national study by Cylus and Papanicolas [24] does look at the impact of variables such as age, education, income, and health status, but because they use data from a single year (2008), they are not able to examine changes over time.

### Policy implications

The main finding from the first part of the article is that, while aggregate per capita OOP spending (in PPP per capita) varies greatly across countries, there is a slow but steady trend towards convergence. Those countries with historically low spending levels (France, Netherlands, the U.K.) have had relatively high growth rates, while the countries with the highest spending (Switzerland, the U.S., Norway) have shown the lowest growth. The four countries with OOP spending near the middle (Australia, Canada, Germany, Sweden) also show growth rates in the middle. This indicates some amount of learning from others’ experiences, but also reflects the possibility that the low spending countries recognize they have more policy wiggle room and that the high spending countries recognize they may have reached the maximum OOP burden of what can be shifted to consumers.

The key finding from the second part of the article is that perceptions of the reduced access to care posed by OOP spending are influenced by three factors: per capita spending, recent changes in OOP requirements, and the existence of effective safety mechanisms. It is perhaps not surprising that the two countries with the highest per capita OOP spending (Switzerland and the U.S.) show the highest levels of perceived cost-related access barrier in 2016. Similarly, the country with the lowest aggregate spending level, the U.K., has the lowest rate of perceived barriers to access. But these patterns do not always hold; France also has low OOP spending, but the third highest rate of perceived cost-related access barriers in 2016. Conversely, Norway has high OOP spending, but low perceived cost related access barriers. This shows that there are more factors driving perceived barriers to accessing care.

Indeed, a second factor driving perceived reductions in access in some countries is recent changes to cost sharing requirements. People often evaluate their welfare based not so much on what they have, but rather, on changes to the status quo [25]. The Netherlands, a country with historically low OOP spending, provides a case in point. Deductibles were introduced in 2008, but they more than doubled in the two-year period between 2011 and 2013. Even though the amounts were low compared to countries like Switzerland and the U.S., this fast rise coincided with more than one-fifth of the population reporting cost-related access barriers in 2013 compared to just 6% three years earlier. As deductibles stabilized, the reported access problems returned to historic levels (8%) by 2016. Somewhat analogously, France, a country with very low aggregate OOP spending, reports high and growing perceived access barriers, which are likely due, in part, to increases in co-payments. Thus, individuals perceive access barriers based not only how much they have to pay OOP, but also according to what they are accustomed to paying.

A third factor that appears to affect perceived barriers: the existence of effective policies to counter high individual OOP burdens. We distinguish two kinds: safety-net mechanisms and policies aiming to more equally spread the OOP burden. It is noteworthy that even though Switzerland is, and has been for some time, an outlier with regard to aggregate OOP spending – with aggregate per capita amounts 60% higher than the U.S. – its population reports far fewer access barriers. This is due to several factors. First, Switzerland has achieved universal coverage of its population since 1996. Second, children and maternity services are exempted from most cost sharing requirements. Finally, annual maximums on coinsurance payments exist. In contrast, the U.S. has far weaker protection mechanisms, with a large proportion of the population uninsured and very high deductibles in many health plans. Using Norway as an example to follow, policies could be aimed at more equitably spreading OOP among the population – such as coupling relatively high co-payments with relatively low out-of-pocket maximums and generous exceptions and protections. The U.S. movement towards very high deductibles (without protection mechanisms) has a large impact – both economic and health – on vulnerable groups.

The two most common complaints about relying on OOP requirements are related to each other: they provide a barrier to receiving needed care, and they are regressive. Except for the U.S., where there continues to be a sizable contingent of uninsured persons, all the countries included in this study have formulated policies to help shield most of the economically vulnerable. Those who are protected through lower or zero OOP requirements include low-income persons (all countries), children, those with high expenditures, and people with particular diseases, and older adults. Some of these mechanisms appear to be more successful than others, judging from the large amount of variation in perceived cost-related access problems shown in Fig. 3.

High deductibles are particularly problematic for those with lower incomes and those who are sicker. Not only are they regressive by nature, but they can be a formidable barrier to care. If people do not expect to meet their deductibles during the year, they will behave as though they are uninsured, and repeatedly studies have shown that such economic barriers result in receipt of fewer needed services and lack of compliance with prescription drug regimens [26]. The two countries that rely most heavily on deductibles – the U.S. and Switzerland – showed the greatest access barriers in Fig. 3, and as noted the Netherlands also exhibited such barriers when it substantially increased them.

Of course, other cost containment mechanisms are available to control costs. “Supply side cost-sharing” policies such as global budgeting, supply and technology controls, managed care, and incentive reimbursement of providers are used in many countries [27, 28], and have the potential to improve efficiency and equity. Nevertheless, as OOP costs are an essential component of financing sources in all countries, it is important to design policies that limit as far as possible, their negative consequences for patients.

## Conclusions

Since OOP requirements are part of health system financing in all countries, it behooves policy makers to find ways to make them less of an access barrier and less regressive. This requires a better understanding of the root causes of OOP spending. However, in most countries OOP data is not gathered in disaggregated fashion, i.e. broken down into cost sharing and direct payments. The latter relate to the coverage dimensions of uninsurance (“breadth”) and underinsurance (“scope”, i.e. lacking benefits) and in many cases these dimensions may be underestimated. Moreover, more granular data need to be available to allow for comparisons between consumer groups (e.g. income-level or health status) to better understand the differential impacts of OOP policies and spending across populations. Statistical agencies should therefore focus their efforts on disaggregating OOP data, so that future research can: (1) better analyze OOP spending, (2) connect these to actual policies on the ground, and (3) suggest effective solutions to policy makers.

## Appendix

**Table 3:** Key Out-of-Pocket Payment Requirements in the most Recent Year, by Country. This annex contains a table presenting a full description of all out-of-pocket payment requirements in the ten high-income countries studied in this paper

	Depth				Scope	Breadth
	Deductibles	Co-insurance and co-payments	Extra-billing and reference prices	Protection mechanisms		
Australia^a^	none	- specialist ambulatory care: 15% co-insurance - prescriptions: co-payment: AUD38.30 - hospital care: 25% co-insurance at private hospitals	- physicians may bill above fee schedule - private hospitals may bill above fee schedule	- prescriptions: reduced co-payment for low-income and children: AUD6.20 + cap at AUD372; others: cap at AUD1,476 after which low-income co-payment applies. - ambulatory care: co-insurance cap AUD447; cap on OOP for extra billing at AUD648 for low income and children and AUD2,030 for others (80% of costs are covered after reaching the cap)	excluded services: adult dental care, OTC drugs VHI exists for excluded services and private hospital care	universal coverage, + VHI held by about 50%
Canada	- for prescriptions (depending on province)	- prescriptions (depending on province): co-payment or co-insurance	- not allowed	- prescriptions: provincial regulations determine OOP caps and exemptions - low income: various provincial programs cover OOP costs - tax credits for individuals whose medical expenses exceed 3% of annual income	Excluded services (depending on province): prescriptions, vision, dental care, home care, rehabilitation, medical devices/aids VHI exists for many of the excluded services	universal coverage, + two thirds with VHI
England^b^	none	- prescriptions: co-payment GBP8.40 - dental care: GBP19.70, 53.90, or 233.70, depending on type of service	- none	-exemptions: children, low income, certain diseases, + for prescriptions and eye tests also aged 60+ - annual cap on prescriptions co-payment (prepayment certificate): GBP104	Excluded services: - private services - vision aids, eye tests	universal coverage
France	none	- physician visits: 30% co-insurance + €1 co-payment per visit and lab test/x-ray - prescriptions: 15–100% co-insurance + €0.5 co-payment/pack - inpatient care: co-insurance: 20% + co-payment €18/day - dental care: co-insurance (30%) + co-payment €1 - additional co-payment for expensive care (>€120): €18 (once per visit or stay)	- 20% of physicians bill above fee schedule - reference prices exist for dental care, glasses, dentures, hearing aids (covering as little as 10% of costs)	- children exempted from co-payments - patients with one of 32 severe chronic diseases exempted from co-insurance - low income (10% of population) receive free VHI, free vision care, free dental care - complementary VHI covers co-insurance - hospital co-payment limited to 31 days - €50 caps on co-payments each for physician visits, prescriptions, and dental care	none	universal coverage, + 95% with complementary VHI
Germany^c^	None	- prescriptions and medical aids: 10% co-insurance (min €5, max €10) per prescription - Hospital co-payment: €10 per day - Home nursing, physiotherapy, etc.: 10% co-insurance + €10 per prescription	- Reference prices exist for crowns and dentures (covering about 50% of normal treatment), prescriptions, and medical aids.	Exemption: Children under age 18; Maximum cost-sharing (does not apply to OOP above reference prices): 2% of annual income (1% for patients with chronic conditions)	excluded services: OTC drugs, certain services of uncertain benefit or unfavourable cost-effectiveness.	universal coverage; 10% covered by substitutive VHI
Netherlands	- €385 for all services except primary care	- 20-25% co-insurance for non-contracted care (only for benefits in-kind insurance policies)	- prescriptions: OOP above reference price - medical devices and aids: OOP above reference prices	deductible exemption: children < 18, maternal care, integrated care programs	excluded services: adult dental care, certain prescriptions (statins, ASS, benzos), physiotherapy	universal coverage
Norway^d^	None	co-payments: - primary care: NOK 152/201 - specialist care: NOK 345 - physiotherapists: NOK 98–300 - psychologist: NOK 345–1035 - dental care for 19–20 years: 25% in co-payment if services are provided by public providers - prescriptions: 39% of full price if on the “blue list” (max NOK 520/prescription) - radiology: NOK 245/exam - lab test: NOK 54	- extra billing of services/materials used that are excluded from statutory coverage (this is not included in cap 1 or 2) such as bandage, consumables	- children under age 16 exempted from co-payments, up to 18 years for dental care - Cap 1: max 2205 NOK during a calendar year covering primary and specialist care, radiology and “blue list” prescriptions - Cap 2: max 1990 NOK during a calendar year covering physiotherapists, some dental care for adults (predefined conditions), some rehab, and treatment abroad when referred by Oslo university hospital - tax deductions for patients with medical expenses above NOK 5880	excluded services: adult dental care (with some exceptions for a few predefined conditions), prescription drugs not covered by the “blue list”, services provided by non-contracted providers, services provided or devices/materials used that are excluded from statutory coverage (e.g. bandages, consumables)	universal coverage
Sweden^e^	- prescriptions: 1100 SEK (for adults> 18) - dental care: 3000 SEK (for adults > 22)	co-payments (varying across the 21 county councils): - primary care: 120–200 SEK per physician visit - specialist care: 200–350 SEK per physician visit (reduced if referred from primary care) - hospitalization: 100 SEK/day for adults> 19 - medical devices/aids: co-payment for different types of devices/aids co-insurance (determined at national level): - prescriptions: 50% between 1100 and 2100 SEK, 25% between 2100 and 3900 SEK, 10% between 3900 and 5400 SEK (max 2200 in co-payments for each 12 month period) - dental care: 50% between reference prices of 3000 and 15,000 SEK, 15% for reference prices above 15,000 SEK (applies for each 12 month period)	- dental care: OOP above reference prices - medical devices/aids: OOP above reference prices; extra billing for medical devices/aids not covered in some county councils (e.g. multifocal lenses in cataract surgery, advanced hearing aids)	General exemptions: - children and young adults < 22 for dental care, < 18 for prescription drugs, < 20 for health services Two separate co-payment caps for each 12 month period: - visits to primary and outpatient specialist care combined: 1100 SEK - prescription drugs: 2200 SEK	excluded services: certain medicines, dental services and aids with uncertain benefits and/or poor cost-effectiveness; services provided by non-contracted private providers; vision aids including eye test	universal coverage
Switzerland	all services: min. CHF300 – max CHF2500	- Co-insurance 10% of all costs above deductible; - Hospital co-payment: CHF 15 / day	- medical aids: patients pay OOP above reference price	- Children (< 19 y): no deductible (or voluntary between CHF 100 and CHF 600);- Maximum for co-insurance: CHF700/year (Children: CHF350/year) - exemptions for preventive and maternal care	excluded services: adult dental care, OTC drugs, psychotherapy performed by independent psychologists, vision aids	Universal coverage
US	Employer plans - average: US$1318 Medicare: - hospital care: US$1288 - physician services: US$166 - drugs: varies by plan	Employer plans: - average co-insurance: 18–19% primary, specialist and hospital care - average co-payment: US$24 (primary), US$37 (specialist), US$308 (hospital) - prescriptions: co-insurance 17–32%, co-payment: US$11–93 Medicare: - hospital care: no co-payment for first 60 days, US$322/day until day 90 - physician services: 20% co-insurance - drugs: varies by plan	- usually not allowed	Employer plans: - median cap on user charges: US$3000–3999	Usually excluded: dental care and vision care	8.6% uninsured

^a^AUD amounts refer to 2016 values; refs: https://www.humanservices.gov.au/customer/enablers/2016-medicare-safety-net-thresholds; http://www.pbs.gov.au/info/healthpro/explanatory-notes/front/fee; http://www.commonwealthfund.org/~/media/files/publications/fund-report/2016/jan/1857_mossialos_intl_profiles_2015_v7.pdf

^b^Values refer to 2016

^c^OOP requirements refer to SHI system; requirements for substitute VHI differ

^d^Values refer to 2015, ref.: http://www.commonwealthfund.org/~/media/files/publications/fund-report/2016/jan/1857_mossialos_intl_profiles_2015_v7.pdf

^e^Values refer to 2011

**Table 4:** Changes in Out-of-Pocket Payment Requirements Over 10-Year Period, by Country. This annex contains a table with a full description of the changes in out-of-pocket requirements over a 10-year period in the ten high-income countries studied in this paper

	Depth				Scope	Breadth
	Deductibles	Co-insurance and co-payments	Extra-billing and reference prices	Protection mechanisms		
Australia	-no change	2005: pharmaceutical co-payments increased to AUD28.60 2015, 2016: increase in pharmaceutical co-payments	- annual changes related to decisions of physicians	- 2005: incentive payment to GPs who do not extra bill - annual increase in co-insurance caps - 2016: introduction of cap on OOP for extra billing	- minimal changes	- no change
Canada	- no change	- provincial level changes for prescriptions	- no change	- provincial level changes for low-income and elderly caps/exemptions	- no federal level change but provincial level changes	- no change
England	- no change	prescriptions: - annual increase of co-payments by GBP0.10–0.20 dental care: - 2006: reduction of co-payments (maximum reduced from GBP384 to 189) - since then regular increase	- no change	- regular increase of prescriptions cap	- no change	- no change
France	- no change	2005: introduction of €1 co-payments for physician visits, lab tests, x-rays 2006: introduction of €18 co-payment for expensive care (>€120) 2008: introduction of €0.5 co-payment for prescriptions 2013: increase of daily co-payment for hospital care from €16 to €18	- changes related to choices of physicians	2005 and 2008: introduction of €50 caps on co-payments for each type of service	- minimal changes	- no change; continuous growth of complementary VHI coverage
Germany	- no change	2013: €10 per physician visit discontinued	2005: introduction of reference price system for crowns and dentures	- no change	- minimal changes	since 2007: mandatory insurance
Netherlands	2008: €150 deductible introduced 2009–2016: annual increase up to €385	since 2010: emergence of co-insurance for non-contracted providers	- no change	2014: several compensations for chronically ill were abolished (e.g. partial compensation for the mandatory deductible), but municipalities may provide such compensations now.	Many exclusions and some inclusions, e.g.: 2006: exclusion of adult dental care 2007: inclusion of psychotherapy (severe cases), first IVF (of max. 3) 2009: exclusion of benzos, statins 2009 exclusion of walkers 2011: exclusion of dental care for 18–22 y/o 2011/12: reductions in physical therapy 2012: exclusion of gastric acid blockers 2013: exclusion of simple walking aids 2013–14: inclusion of quit smoking and dietary advice	- no change
Norway	- no change	- small changes to co-payment amounts, slowly increasing	- no change	- annual revision of co-payment cap, slowly increasing	- minimal changes	- no change
Sweden	- no change	- small increases to co-payment amounts for outpatient specialist care without referral in several county councils	- no change	2008: reduced co-insurance for dental care above high cost threshold 2012: OOP caps increase: - prescriptions: from SEK 1800 to 2200 - outpatient primary and specialist care: from SEK 900 to 1100 2016: introduction of general exemption from co-payments for prescription drugs for children under 18	2009: prescription drug coverage restricted to lowest cost generic 2013: minor increase in scope of subsidies in dental care to elderly	- no change
Switzerland	- 2005: max deductible is increased from CHF1500 to CHF2500 - Proportion of insured opting for deductible of CHF2500 increased from around 5% in 2005 to 19% in 2014	2011: hospital co-payment is increased from CHF10 to: CHF 15 / day; 2006: Co-insurance is increased to 20% for brand drugs if a cheaper generic is available;	-no change	- no change	2011: vision aids excluded from coverage; 2012: alternative and complementary medicine included in benefits catalogue	- no change
US	Employer plans: - average deductible increase from US$602 in 2005 to US$1318 in 2015 Medicare: - annual small increase in deductibles	Employer plans: - co-insurance, co-payments relatively stable Medicare: - annual small increase in co-payments	- no change	Employer plans: - annual 6% increase of proportion of insured with cost-sharing cap of ≥US$3000 (33% in 2005, 59%)	- dental care and vision care excluded in increasing proportion of plans	- coverage among the adult population increased from 82.9% in 2013 to 89% in 2016 [19]

## Abbreviation

OOP: Out-of-Pocket
